# Male breast metastasis of ureteral cancer: a case report

**DOI:** 10.1186/s40792-020-00804-1

**Published:** 2020-03-30

**Authors:** Yoshitaka Ishikawa, Isao Tabei, Atsushi Fushimi, Azusa Fuke, Chikako Sekine, Tomoyoshi Okamoto, Hiroshi Takeyama

**Affiliations:** 1 Moriya Keiyu Hospital Department of Surgery, 980-1 Tatsuzawa, Moriya-shi, Ibaraki, 302-0118 Japan; 2 The Jikei University School of Medicine Daisan Hospital Department of Surgery, 4-11-1 Izumihoncho, Komae-shi, Tokyo, 201-8601 Japan; 3 The Jikei University School of Medicine Hospital Division of Breast Thyroid & Endocrine Surgery, 3-19-18 Nishishimbashi, Minato-ku, Tokyo, 105-8471 Japan

**Keywords:** Male breast cancer, Breast metastasis, Extra-mammary, Intraductal lesion, Ureteral cancer

## Abstract

**Background:**

Breast metastasis from extra-mammary primary tumors is extremely rare. We recently experienced a rare case of a male breast metastasis of ureteral cancer and will provide a case report.

**Case presentation:**

A 74-year-old man developed a ureteral cancer and underwent left nephroureterectomy with lymph node dissection. Although enlarged abdominal lymph nodes did not disappear completely after chemoradiotherapy, further extensive therapy was not performed. A mass just below the nipple of his right breast was acknowledged and he visited our department. Histological diagnosis was invasive carcinoma. It was initially diagnosed as a primary breast cancer, and he underwent a mastectomy and a sentinel lymph node biopsy. There was no intraductal lesion and the border of the tumor was clear. It was very similar to that of the previous ureteral cancer. The final diagnosis was breast metastasis of ureteral cancer rather than primary breast cancer. The postoperative course was good, but multiple lung metastases appeared 2 months after surgery. He eventually died of cancerous lymphangiopathy.

**Conclusion:**

It is important to accurately diagnose primary breast cancer or breast metastasis so as not to cause extra-invasion, but it was considered difficult to make a complete preoperative diagnosis.

## Background

The incidence of breast metastasis from extra-mammary primary tumors ranges from 0.3 to 2.7% [1]. Malignant melanoma, lung cancer, gynecological cancers, and hematologic malignancies are some of the most common among all malignancies that have been described as metastasizing to the breast [2]. However, there have been no reports of breast metastasis originated from ureteral cancer. We recently experienced a rare case of a male breast metastasis of ureteral cancer and will provide a case report with a review of the relevant literature.

## Case presentation

**Fig. 1:**
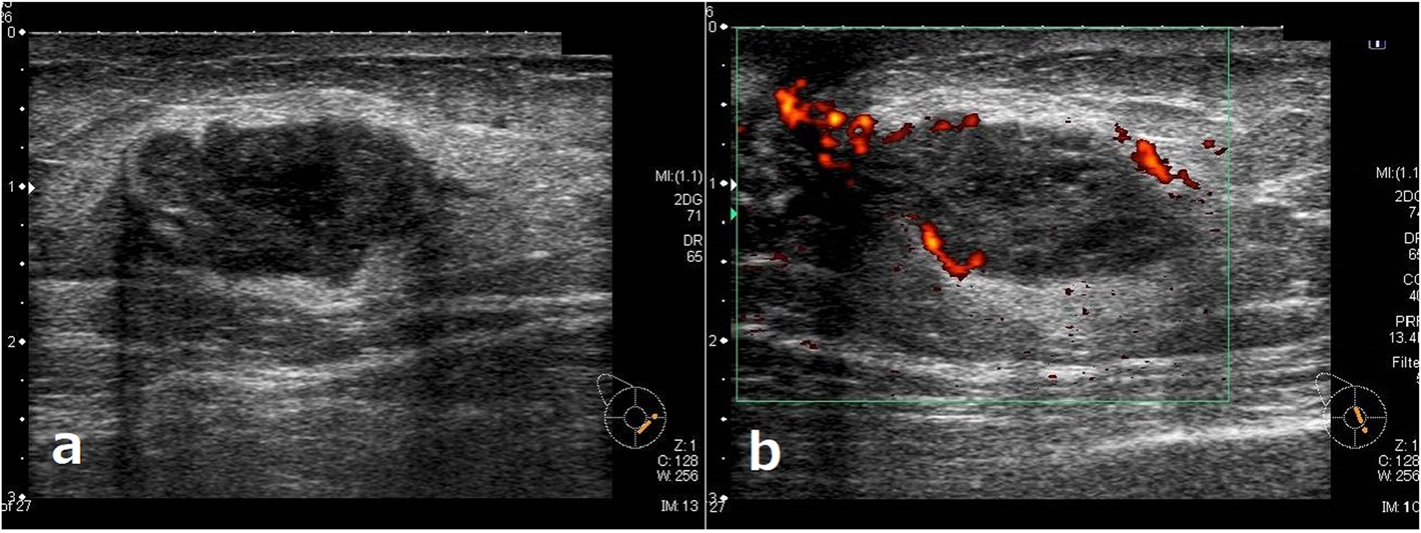
Breast ultrasonography. Legend: Breast ultrasonography showed an irregular, rough, internally inhomogeneous hypoechoic mass measuring 32 × 35 mm in the center of the right breast right beneath the nipple (**a**, **b**)

**Fig. 2:**
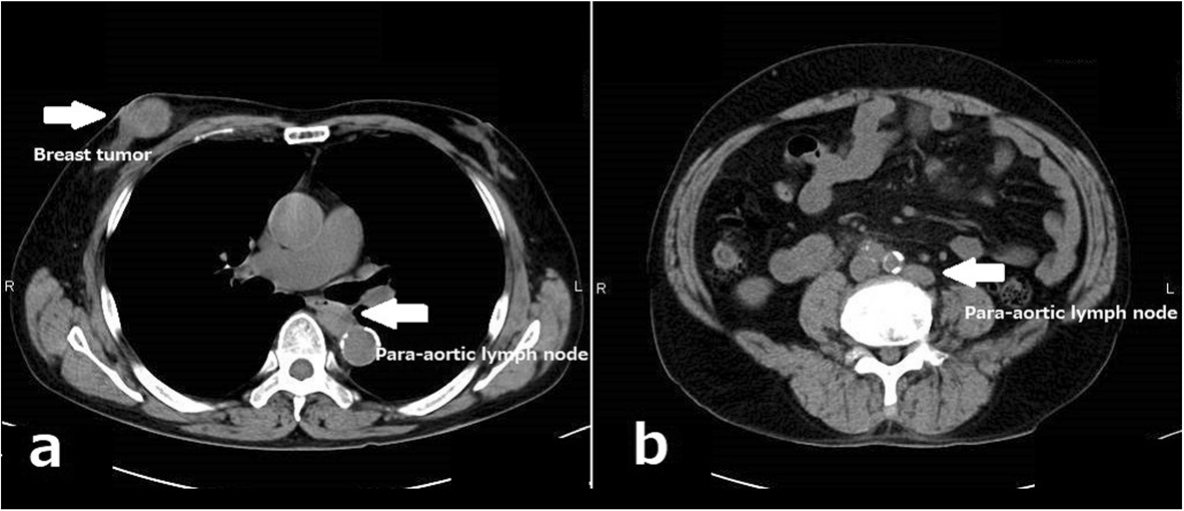
Chest and abdominal computed tomography. Legend: Computed tomography showed the right breast tumor (**a**) and the para-aortic lymph nodes enlargement which had not changed for several months (**a**, **b**)

**Fig. 3:**
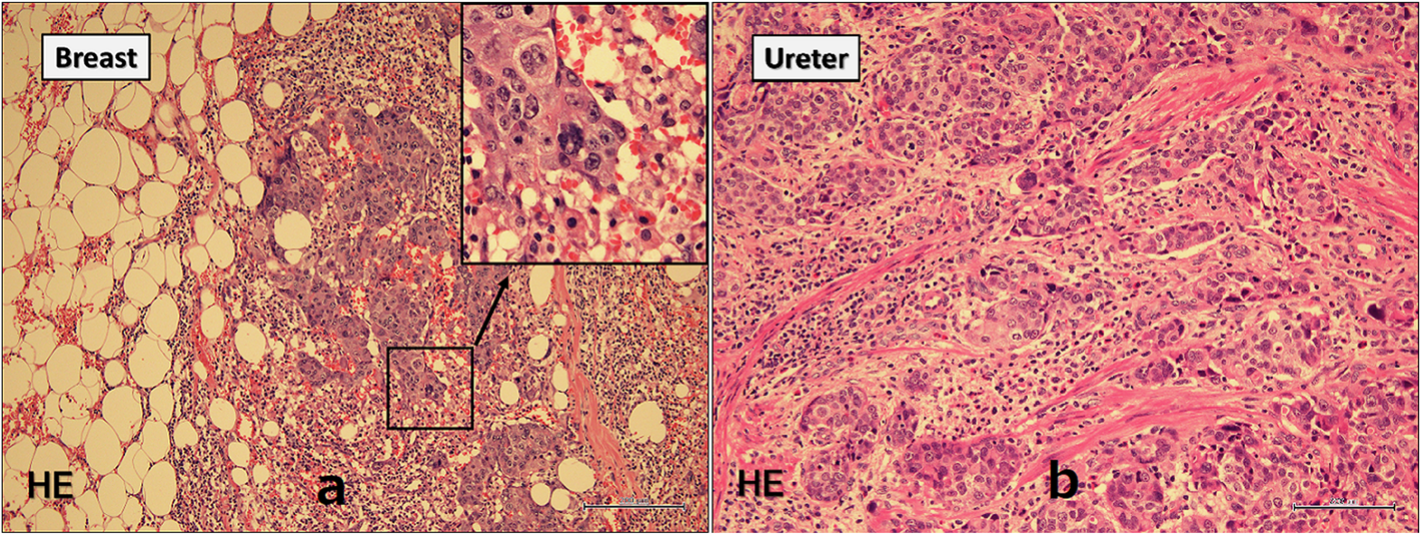
Permanent pathology of the breast tumor. Legend: Tissue images showed no intraductal lesion and the border of the tumor was clear (**a**). It was very similar to that of the previous ureteral cancer (**b**)

**Fig. 4:**
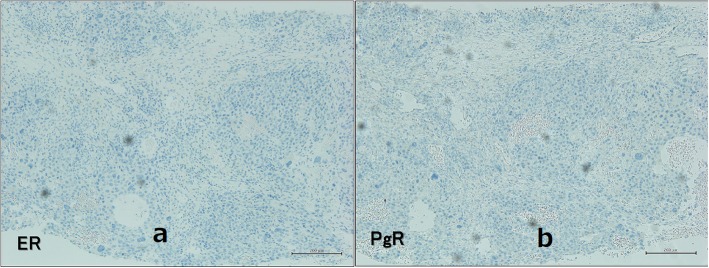
Imunohistochemical images of the breast tumor. Legend: Immunohistochemical images showed negative results for estrogen receptor (**a**) and progesterone receptor (**b**)

A 74-year-old man developed a ureteral cancer and underwent left nephroureterectomy with lymph node dissection at the Department of Urology of The Jikei University School of Medicine Daisan Hospital in September 2013. After radical surgery, he repeatedly received chemoradiotherapy to treat for para-aortic lymph nodes recurrence. Although enlarged abdominal lymph nodes did not disappear completely, further extensive therapy was not performed, and active surveillance was followed for these lymph nodes in consideration of his age and renal function since September 2015. He had no history of malignancy other than ureteral cancer, no breast disease, and no family history of carcinoma. A mass just below the nipple of his right breast was acknowledged in February 2016, and he visited our department in March. On primary clinical examination, a hard 3-cm mass with good mobility was palpated just below the right nipple. Breast ultrasonography (US) revealed an irregular, rough, internally inhomogeneous hypoechoic mass measuring 32 × 35 mm in the center of the right breast right beneath the nipple (Fig. 1). A tissue needle biopsy was performed, and histological diagnosis was invasive carcinoma, which showed negative results for estrogen receptor and progesterone receptor. The mass was 1 cm larger in 1 month. Although the intraductal lesion around the carcinoma was not clear and the tissue similarity to that of the previous ureteral cancer was considered, few findings actively suggested breast metastasis of ureteral cancer, and it was initially diagnosed as a primary breast cancer. The abdominal US showed no liver metastases. Chest and abdominal computed tomography (CT) showed no axillary lymph node enlargement and no distant metastasis, other than the already defined para-aortic lymph nodes enlargement observed in previous CT (Fig. 2). Preoperative chemotherapy was taken into consideration, but the patient preferred surgery alone. He underwent a mastectomy and a sentinel lymph node biopsy in April 2016. Gross pathology showed a 45 × 30 × 55 mm nodular lesion. The tumor cells had high nuclear atypia, and the mitotic figures were extremely prominent. There was no intraductal lesion and the border of the tumor was clear. It was very similar to that of the previous ureteral cancer (Fig. 3). Additional immunohistochemical examination was performed, which showed negative results for mammaglobin, GCDFP-15, estrogen receptor, and progesterone receptor (Fig 4). The histocytological and immunohistochemical findings led to the final diagnosis of breast metastasis of ureteral cancer rather than primary breast cancer. The postoperative course was good, but multiple lung metastases appeared 2 months after surgery. He eventually died of cancerous lymphangiopathy in July 2016.

## Discussion

Breast cancer is one of the most common malignancies. Worldwide, 2.1 million newly diagnosed female breast cancer cases (11.6% of the total cancer cases) were reported in 2018, accounting for almost 1 in 4 cancer cases among women [3]. But the incidence of breast metastasis from extra-mammary primary neoplasms is very rare. The breast as a metastatic site is reported to ranges from 0.3 to 2.7% of all breast malignancies [1]. Some of the common malignant tumors showing breast as a site of metastasis include melanoma (29.8%), lung cancer (16.4%), gynecological cancers (12.7%), and hematologic malignancies (8.4%) [2]. Furthermore, the incidence of malignancy of the male breast is about 1% of all breast malignancies [4]. There have been no reports of breast metastasis of ureteral cancer described in the literature in females as well as males. Our case is probably the first to describe a metastatic tumor with a ureteral carcinoma origin in the breast of a male patient.

Ureteral cancer refers to any malignancies that arise from the urothelial lining of the urinary tract, from the calyceal system to the distal ureter. It is a relatively uncommon entity, accounting for 5–7 % of all renal tumors and 5–10 % of all urothelial tumors, with an estimated annual incidence of 1–2 cases per 100,000 [5]. The common metastatic sites of ureteral cancer are the lung, distant lymph nodes, liver, and bone. The presence of metastasis is associated with poor prognosis [6, 7].

While the characteristic clinical and laboratory findings of breast metastasis remain unclear, in many instances, they include tumors with good mobility and relatively distinct borders [1]. According to a report about breast metastases, solitary or multiple round-to-oval masses with distinct borders are delineated on mammography; of these, 10% exhibit micro-calcification within the tumor [8]. On breast US, most breast metastases are described as round-to-oval hypoechoic masses with posterior acoustic enhancement and clearly delineated or smooth and distinct borders [8].

In our case, tissue biopsy could not rule out the breast metastasis of ureteral cancer, but there were few other findings positively suggesting it. Moreover, previous ureteral cancer did not deteriorate during follow-up without treatment, and it was thought that it progressed very slowly and was not related to a rapidly growing breast mass. Therefore, we preoperatively diagnosed as primary breast cancer.

Histopathologically, images of tumors with a distinct border and no calcification around normal mammary glands or no characteristics of intraductal carcinoma are findings that strongly suggest breast metastasis of malignant tumors of other organs [9]. Moreover, immunohistochemistry can be very valuable when trying to differentiate between a primary cancer originating in the breast and a metastasis to the breast and identify the primary organ of malignant tumors [10, 11]. Although histological and immunohistochemical examination is considered feasible to diagnose primary cancer or metastatic tumor, it is very difficult to make preoperative diagnosis by tissue biopsy, like our case. The postoperative pathological diagnosis is also not easy. In our case, there was histologically no intraductal lesion and the border of the tumor was clear; however, it took multiple pathologists to finally diagnose breast tumor as metastasis of ureteral cancer because breast metastases are extremely rare.

Patients with the breast metastasis from the extra-mammary origin have a poor prognosis. In a series of 169 patients with confirmed metastases to the breast from extra-mammary solid organ primary tumors, it was found that the median survival time from the diagnosis of breast metastasis was 10 months [12]. On a univariate analysis, a significantly higher survival rate was observed in patients who underwent surgical resection for breast metastases. On multivariate analysis, those individuals who did not undergo surgery were 88% more likely to succumb than those who underwent surgery [12].

## Conclusion

We reported an extremely rare case of breast metastasis from ureteral cancer. In such cases, it is important to accurately diagnose primary breast cancer or breast metastasis so as not to cause extra-invasion, but it was considered difficult to make a complete preoperative diagnosis.

## Data Availability

There is no available data and materials to be shared.

## References

[1] Lee SK, Kim WW, Kim SH, Hur SM, Kim S, Choi JH (2010). Characteristics of metastasis in the breast from extramammary malignancies. J Surg Oncol..

[2] Koch A, Richter-Marot A, Wissler MP, Baratte A, Mathelin C (2013). Mammary metastasis of extramammary cancers: current knowledge and diagnostic difficulties. Gynécol Obstét Fertil..

[3] Bray F, Ferlay J, Soerjomataram I, Siegel RL, Torre LA, Jemal A (2018). Global cancer statistics 2018: GLOBOCAN estimates of incidence and mortality worldwide for 36 cancers in 185 countries. CA Cancer J Clin.

[4] Ferzoco RM, Ruddy KJ (2016). The epidemiology of male breast cancer. Curr Oncol Rep.

[5] Siegel RL, Miller KD, Jemal A (2016). Cancer statistics. CA Cancer J Clin..

[6] Kondo T, Nakazawa H, Ito F, Hashimoto Y, Toma H, Tanabe K (2007). Primary site and incidence of lymph node metastases in urothelial carcinoma of upper urinary tract. Urology..

[7] Inokuchi J, Naito S, Fujimoto H, Hara T, Sakura M, Nishiyama H (2016). Impact of multimodal treatment on prognosis for patients with metastatic upper urinary tract urothelial cancer: subanalysis of the multi-institutional nationwide case series study of the Japanese Urological Association. Int J Urol..

[8] Surov A, Fiedler E, Holzhausen HJ, Ruschke K, Schmoll HJ, Spielmann RP (2011). Metastases to the breast from non-mammary malignancies: primary tumors, prevalence, clinical signs, and radiological features. Acad Radiol..

[9] Lee AH (2007). The histological diagnosis of metastases to the breast from extramammary malignancies. J Clin Pathol.

[10] Schurch W, Lamoureux E, Lefebvre R, Fauteux JP (1980). Solitary breast metastasis: first manifestation of an occult carcinoid of the ileum. Virchows Arch A Path Anat Histol..

[11] Solaini L, Bianchi A, Filippini L, Lucini L, Simoncini E, Ragni F (2014). A mammary nodule mimicking breast cancer. Int Surg..

[12] Williams SA, Ehlers RA, Hunt KK, Yi M, Kuerer HM, Singletary SE (2007). Metastases to the breast from nonbreast solid neoplasms: presentation and determinants of survival. Cancer..

